# Real-world effectiveness of a social-psychological intervention translated from controlled trials to classrooms

**DOI:** 10.1038/s41539-022-00135-w

**Published:** 2022-08-29

**Authors:** Patricia Chen, Dennis W. H. Teo, Daniel X. Y. Foo, Holly A. Derry, Benjamin T. Hayward, Kyle W. Schulz, Caitlin Hayward, Timothy A. McKay, Desmond C. Ong

**Affiliations:** 1 Department of Psychology, National University of Singapore, Singapore, Singapore; 2 Institute for Applied Learning Sciences and Educational Technology, National University of, Singapore, Singapore; 3 Department of Information Systems and Analytics, National University of Singapore, Singapore, Singapore; 4 The Center for Academic Innovation, University of Michigan, Ann Arbor, MI USA; 5 Departments of Physics and Astronomy, College of Literature, Science, and the Arts, and School of Education, University of Michigan, Ann Arbor, MI USA; 6 Department of Psychology, University of Texas at Austin, Texas, USA

**Keywords:** Education, Human behaviour

## Abstract

Social-psychological interventions have raised the learning and performance of students in rigorous efficacy trials. Yet, after they are distributed “in the wild” for students to self-administer, there has been little research following up on their translational effectiveness. We used cutting-edge educational technology to tailor, scale up, and track a previously-validated Strategic Resource Use intervention among 12,065 college students in 14 STEM and Economics classes. Students who self-administered this “Exam Playbook” benefitted by an average of 2.17 percentage points (i.e., a standardized effect size of 0.18), compared to non-users. This effect size was 1.65 percentage points when controlling for college entrance exam scores and 1.75 [−1.88] for adding [dropping] the Exam Playbook in stratified matching analyses. Average benefits differed in magnitude by the conduciveness of the class climate (including peer norms and incentives), gender, first-generation status, as well as how often and how early they used the intervention. These findings on how, when, and who naturally adopts these resources address a need to improve prediction, translation, and scalability of social-psychological intervention benefits.

## Introduction

“Psychological interventions are done with people, not on people, and these people live in dynamic and diverse social contexts. To predict intervention effects and to advance theory, application, and replicability, we need to understand where and when people will accept the way of thinking put forth by the intervention and be able to use it in their lives to good effect and where and when they will not.” Walton & Yeager (2020, p. 224^1^).

Policy-makers, educators, psychologists, economists, and school administrators go to painstaking lengths to create interventions to improve student achievement. Some interventions take the form of structural and curricular changes^2^, whereas others are more social-psychological in nature—targeting important mental, emotional, motivational, or social mechanisms of learning, and are often delivered directly to the individual student^3,4^. Successful social-psychological interventions have effectively raised the learning, performance, and well-being of tens of thousands of students across the achievement spectrum in rigorous double-blind, randomized controlled trials (RCTs)^5–10^.

The end goal of developing and rigorously testing these psychological interventions in RCTs is to provide them as effective psychological resources to students, parents, and educators. While there are multiple ways to scale social-psychological interventions (such as offering them to teachers to use as pedagogical tools, conducting school-wide workshops, or incorporating intervention content into the curriculum), social-psychological interventions that students can self-administer to help themselves are especially important and relevant in secondary, post-secondary, and online education, where learning is largely self-regulated.

Once tried-and-tested social-psychological interventions are widely distributed “into the wild” for students to self-administer, does their use still predict academic achievement? What kinds of students take up these interventions on their own, and how effectively do they use the resources? Under what conditions are they more or less effective? These are important scientific questions about the translational effectiveness of self-administered interventions (including translational effect sizes, user uptake, and heterogeneity).

To our knowledge, unlike school-wide program evaluations, there are no rigorous, large-scale naturalistic examinations of such *effectiveness* of student-level social, affective, or motivational interventions, after they are distributed for students to adopt on their own. There already exists many examples of efficacious social-psychological interventions^3^, established through gold-standard laboratory and classroom experiments—such as the Strategic Resource Use intervention, which was tested in two RCTs with relatively large effect sizes on course grades^6^; the values affirmation intervention, which was replicated in many RCTs across different age groups and school sites^11,12^; the social-belonging intervention, which produced performance and health benefits among minorities across multiple RCTs, including a large field experiment across 21 colleges and universities^1,9^; and the growth mindset intervention, which robustly replicated over many experiments and was recently tested in a randomized, controlled trial using a nationally representative sample of 65 U.S. high schools^10^. Some of these interventions have even been tested cross-culturally via Massive Open Online Courses (MOOCs)^13,14^.

These rigorous experiments robustly demonstrated *efficacy*—but speak less to the interventions’ *effectiveness* after being released “into the wild” for learners to adopt and self-administer as a self-regulatory choice. We borrow these terms from the medical and public health literature, where *efficacy* studies establish scientific validity in controlled experiments, whereas *effectiveness* studies test external validity when the intervention is delivered in the real world^15,16^. Compared to efficacy trials, the success of effectiveness studies like ours depend on multiple factors, including but not limited to the efficacy and accessibility of the intervention, along with participants’ receptivity to, willingness to engage in, and effective use of the intervention^15^.

Despite their importance, efficacy trials alone are considered “necessary… but not sufficient for, effectiveness” in the real world (^15^, p. 455), where people have the choice of using or not using any resource amongst many others available. To understand what keeps psychologically wise interventions effective for student learning when they progress from an experimental trial to a freely available resource, scientific inquiry must go beyond controlled experiments^16,17^. Some social-psychological interventions may have been beneficial under relatively controlled conditions when the intervention was imposed upon students, often in a highly structured manner (i.e., students have no choice about which treatment to engage in or when they do so, but are assigned to either a treatment or control, and directed when and how to engage with the materials). But in real-world learning, especially starting from mid- to late-adolescence where learning becomes increasingly self-managed, there is no guarantee that students would make use of a particular intervention among many alternative resources, that students would even use the intervention effectively on their own, or that intervention use would relate to academic performance.

Hence, especially for interventions that will eventually be given to students to use as tools for learning, it is important to conduct naturalistic studies of translational effectiveness that follow-up and complement efficacy-focused experiments. Such studies provide much-needed information about (a) whether self-administration of the intervention relates to academic achievement for most students, (b) under what conditions using the intervention confers more versus less benefits, and (c) how intervention use and benefits may differ across different kinds of learners.

To study these questions, we adapted a previously-validated Strategic Resource Use intervention, which was designed to increase students’ self-reflection about their resource use^6^, into an online app called the “Exam Playbook.” We chose the Strategic Resource Use intervention because it had previously been experimentally tested and found efficacious at raising students’ course grades by an average of one-third of a letter grade in two double-blind RCTs (total *N* = 361; effect size Cohen’s *d* = 0.33 and 0.37) at the same university^6,18^; it was online and self-administered, and therefore could be conveniently modified for testing across a variety of classes; and it benefitted diverse demographic groups of students in prior experiments^6^. Moreover, the university administration was supportive of widely distributing and testing the use of this intervention as a free resource for students.

The Exam Playbook was made user-friendly and engaging for the average student. Similar to its predecessor^6^, the Exam Playbook guided students’ self-reflective resource use when preparing for an upcoming exam. It prompted students to anticipate the format and demands of their upcoming exam, provided a comprehensive checklist of learning resources available in the class, and asked students to select which would be useful in helping them study for the exam. It asked students to explain why each resource they had chosen would be useful to their learning (to make clear the purpose for which they would use the resource), and then to plan out when, where, and how they were going to use the resources chosen (which increases the likelihood of follow-through on their plans^19^).

We used cutting-edge “ECoach” technology, developed within the past 6 years, to innovate on class-level customizations to the Exam Playbook (e.g., resource checklist, distribution timing)—making it highly scalable across diverse class contexts (see Methods for details;^20,21^). Through ECoach, we made the intervention freely available to college students enrolled in 14 large introductory STEM classes across 2 semesters at a large public U.S. university. Students could freely choose and access our intervention, as one of many possible learning resources (behaviors which ECoach tracked). This allowed us to collect behavioral data about who accessed the intervention and when they did so.

Combining the Exam Playbook with ECoach technology, our translational study focused on estimating intervention effectiveness and understanding the factors that can influence intervention effects, when a social-psychological intervention for learning (in this case, the Strategic Resource Use intervention) is released “into the wild,” where students actively manage their own learning^17^. We tested our hypothesis that, on average, we would observe a statistically-significant relation between using the intervention and students’ exam performance across classes, but that it would be smaller in magnitude than the effect sizes observed in the RCTs (*d*s = 0.33, 0.37;^22^). For reference, a difference of 0.2 is considered a large difference in field research on factors that predict educational outcomes, especially when an intervention is low-cost and scalable^10,23,24^. Beyond testing the main effect of the intervention, we planned additional exploratory analyses to investigate heterogeneity by classes and student demographics.

There are multiple benefits to having this translational effectiveness study complement prior efficacy RCTs: One, we can estimate the effectiveness of the intervention when students autonomously self-administer it^16^. Two, we can capture what kinds of students are utilizing the intervention in the real world under realistic learning conditions, and how they are making use of it (e.g., timing, dosage)^25,26^. Three, we supplement the previous RCTs^6^ by showing that an intervention tested on a small scale can be effective when distributed on a large scale. Four, we are able to test possible heterogeneity in intervention-use effectiveness across diverse classes. From a policy standpoint, this study of effectiveness is a crucial step when going from bench to bedside—or from “*controlled trial to classroom*” in the education context—especially for interventions that could be widely distributed to hundreds or thousands of students.

## Results

We examined 12,065 students’ use (versus non-use) of the Exam Playbook across 14 introductory STEM and Economics classes over 2 consecutive (Fall and Winter) semesters. The 7 courses included in each semester were: Introductory Statistics, Introductory Biology, General Chemistry, General Physics, Introductory Programming (for Engineers), Introductory Programming (for Programmers), and Introductory Economics. A breakdown of sample demographics is presented in Supplementary Table 1.

Across both semesters, on average, 43.6% (*SD* = 29.3%; range: 5.6–91.4%) of students in each class engaged with the Exam Playbook at least once. We operationalized a “use” of the Exam Playbook to mean accessing and completing the intervention, which includes: completing the resource checklist, explaining why each resource would be useful, and planning resource use. That is, students had to click through to the end of the intervention to be counted as having used it (Supplementary Note 1 contains further details about how we defined and operationalized “use”). Apart from varying across classes, Exam Playbook use also varied between exams, as a student might choose to use it on one exam but not another. Note that the original intervention was only offered before 2 exams (i.e., 2 doses maximum), but in this translational study, it was offered before all available exams in each class, which could differ by class (with the exception of Physics Exam 4 when it was not offered). Table 1 gives a detailed breakdown of the number of times the Exam Playbook was offered and used on each exam across the different classes.

**Table 1:** Breakdown of the usage of exam playbook.

Course	Semester	Class size	Number of users on any exam	% of students who used the Exam Playbook on:			
				Exam 1	Exam 2	Exam 3	Exam 4
Intro Statistics	Fall	1769	1598 (90.3)	58.4	74.1	80.9	–
	Winter	1796	1642 (91.4)	79.1	72.3	76.7	–
Intro Biology	Fall	560	169 (30.2)	21.1	10.9	6.1	4.8
	Winter	564	307 (54.4)	11.7	44.9	14.0	15.4
General Chemistry	Fall	1342	166 (12.4)	7.8	4.8	0.1	1.2
	Winter	525	85 (16.2)	5.6	11.9	3.3	2.1
General Physics	Fall	684	128 (18.7)	12.6	7.0	1.5	–
	Winter	629	234 (37.2)	8.4	30.5	11.3	–
Intro Programming (Engineers)	Fall	770	334 (43.4)	28.3	32.2	–	–
	Winter	533	346 (64.9)	47.3	52.2	–	–
Intro Programming (Programmers)	Fall	946	613 (64.8)	49.6	55.5	–	–
	Winter	775	536 (69.2)	49.8	59.2	–	–
Intro Economics	Fall	818	99 (12.1)	10.4	4.8	1.7	–
	Winter	354	20 (5.6)	4.2	1.7	1.7	–

*Note*. “Any Exam” gives the number (and percentage) of students who used the Exam Playbook at least once in the class. Numbers for individual exams indicate percentage of students in the class who used the Exam Playbook on that exam. Classes had between 2 to 4 exams.

### Does self-administration of the exam playbook predict exam performance?

We tested the hypothesis that using the Exam Playbook benefits students' exam performance, by comparing the average exam scores of students who used the Exam Playbook at least once in the class with students who did not use the Exam Playbook at all. Following recent recommendations in statistics and psychological science to move toward a focus on effect-size estimation^27,28^, we ran a "mini meta-analysis"^29^ across the 14 classes using a random-effects meta-analysis model^30^, treating each class as a separate "experiment" and with a mind towards analyzing heterogeneity across classes. This allowed us to estimate the generalizability of the effect across classes, as well as the variation due to inter-class differences—both of which are important for understanding how the Exam Playbook can benefit future students in various subjects.

**Fig. 1:**
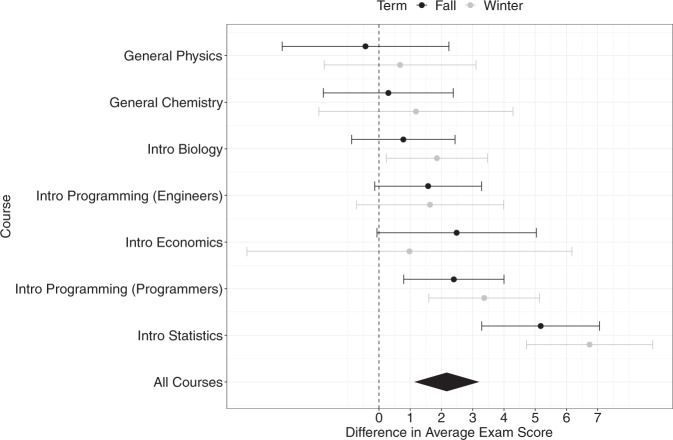
Meta-analysis of the Effect of Using the Exam Playbook. Note. Forest plot summarizing a meta-analysis of the effect of using the Exam Playbook on students’ averaged exam score. Data points represent the effect size for each class in each semester, with error bars representing 95% confidence intervals. The diamond in the last row represents the weighted meta-analytic effect size^30^, and corresponds to a standardized effect size (Cohen’s *d*) of 0.18.

Our meta-analysis, summarized in Fig. 1, revealed that students who used the Exam Playbook in their class scored 2.17 ([95% CI: 1.13, 3.21], *p* < 0.001) percentage points higher than non-users, for their average exam score (normalized and upon 100 percentage points). To put this effect size into context, a 2.17 percentage point difference translates to a standardized difference (Cohen’s *d*) of 0.18—a substantial effect for a free, highly scalable, and self-administered intervention. As mentioned earlier, a difference of 0.2 is considered a large difference in field research on factors that predict educational outcomes, especially for low-cost and scalable interventions^10,23,24^. As Fig. 1 shows, the effect was positive in 13 out of 14 classes, and there was a high correlation of *r* = .87 (*p* = 0.010) between the effect sizes for each class across both semesters.

Two robustness checks further validated these results: One, controlling for students’ college entrance exam scores as a covariate (students in our sample were mostly freshmen who did not yet have college GPA), the overall meta-analytic trend remained consistent: Exam Playbook users scored an average of 1.65 ([0.55, 2.75], Cohen’s *d* = 0.14, *p* = .003) percentage points higher than non-users on their average exam score. We tested demographic factors (gender, race and first-generation status) as potential moderators later in the Results. Two, to supplement our class-level analyses, our results held when we examined Exam Playbook use on performance at the exam-level within class. A mixed-effects meta-analysis (with exam as a fixed effect within each class, and class as a random effect) across all 40 exams observed showed that students who used the Exam Playbook on a given exam scored an average of 2.91 ([1.81, 4.01], Cohen’s *d* = 0.22, *p* < 0.001) percentage points higher than students who did not use the Exam Playbook on a given exam.

### Under what class conditions might the exam playbook be more or less effective?

As shown in Fig. 1, there was substantial heterogeneity in the estimated effect size of using the Exam Playbook across different classes. The average effect size was largest in the Introductory Statistics course (5.18 percentage points in Fall and 6.74 in Winter), which was the exact course for which the original intervention was designed and experimentally tested^6^. Thus, this serves as an assessment of the *effectiveness* of the intervention when made freely available within the same class context (c.f. an RCT-based efficacy effect size of 3.64 and 4.21 percentage points in two studies in^6^).

The other courses allow us to examine the generalization of the Exam Playbook to different class contexts. As a conservative test of the generalizability of Exam Playbook use on exam performance beyond the Introductory Statistics course, we repeated our analyses using only the 6 other courses (12 classes total) excluding Introductory Statistics. On average, using the Exam Playbook still conferred benefits to students in these courses. The meta-analytic effect size was smaller and still significant: students who used the Exam Playbook scored an average of 1.60 ([1.00, 2.19], *d* = 0.13, *p* < 0.001) percentage points higher than non-users. When controlling for college entrance exam scores, we observed a 1.07 percentage points difference ([0.29, 1.85], *d* = 0.09, *p* = 0.007).

After Introductory Statistics, which had the highest use rates and effect sizes, students in the two Introductory Programming courses enjoyed the next-largest average benefits—2.24 percentage points averaged across both semesters and both programming courses (we note that the Introductory Economics course had substantial differences in effect sizes and uptake across Fall and Winter semesters). On the other end of the spectrum, the smallest average effect sizes from using the Exam Playbook were observed in the General Physics and General Chemistry courses (0.12 percentage points averaged across both semesters for General Physics; 0.74 percentage points for General Chemistry).

One plausible reason for such heterogeneity at the class level could be how much the climate of the course supported such strategic resource use, including Exam Playbook use. According to contemporary theorizing about psychological intervention effect heterogeneity, “change requires planting *good seeds* (more adaptive perspectives)… in *fertile soil* (a context with appropriate affordances)” (^1^, emphasis ours). That is, perhaps the Exam Playbook was more useful to students who were in course climates more conducive to the psychology of the Exam Playbook.

Two possible operationalizations of this course climate (at the class-level) are peers’ uptake of the Exam Playbook^10,31^ and teachers’ degree of support toward engaging in the Exam Playbook as a useful learning resource^21^—both of which reflect powerful social norms that could influence students’ engagement with and degree of benefit from the Exam Playbook^1,10,32^.

We fit two separate linear models using (a) the average Exam Playbook usage (by course) and (b) the quantifiable presence/absence of extra course credit offered for engaging in the Exam Playbook, to predict the effect size for each class. Instructors in 4 of the 7 courses (specifically Introductory Statistics, Introductory Biology, Introductory Programming (Programmers), and Introductory Programming (Engineers)) incentivized the use of the Exam Playbook by offering bonus credit to students' final course grade for using it. Importantly, however, these bonuses did not influence our main outcome measure: exam performance.

Indeed, the average Exam Playbook usage in a class (the peer norm) was positively associated with the effect size of using the Exam Playbook (*b* = 2.49 [1.82, 3.16], *d* = 0.20, *p* < 0.001). Similarly, teacher support in the form of course credit incentives offered related to a larger effect size than when it was not offered (*b* = 2.04 [0.25, 3.84], *d* = 0.17, *p* = 0.046).

Could differences in the extensiveness of resources provided or the kinds of resources most students selected to use (such as practice-based versus simple reading and memorization) have explained the variation in effect sizes across classes? Our data did not support either of these possibilities: the number of resources offered varied only slightly among classes (range: 11–15), and the types of resources that students selected the most for use were generally similar across classes (see Supplementary Note 2). Hence, we ruled out that that either of these factors strongly explained class-level heterogeneity.

### Intra-individual changes in exam performance when dropping vs. adopting the exam playbook

One difficulty of observational (effectiveness) studies, compared to experimental (efficacy) studies, is teasing apart the effects of confounding variables. Methods such as matching and difference-in-difference modeling try to control for these effects. We conducted two analyses based on matching, to examine how intra-individual variation in Exam Playbook usage tracked changes in academic performance. We matched students using their background and behavior in the initial portion of the class, and then examined how subsequent behavior tracked exam performance.

In these classes, there were natural variations in Exam Playbook usage. Some students started off not using the Exam Playbook, and picked up (or “adopted”) the Exam Playbook on later exams, while others used the Exam Playbook early on but dropped it later in the class (see Supplementary Table 2 for descriptives). These natural covariations allowed us to assess the average effect of “adopting” and “dropping” the Exam Playbook within individuals. If Exam Playbook usage benefits students’ performance, we should expect their exam performance to covary with students’ Exam Playbook usage patterns—with “adopting” and “dropping” associated with increased and decreased exam performance, respectively.

Using stratified matching^33^, we matched these students on their initial exam performance (the first exam in the class), college entrance scores, gender, race, and first-generation status, and estimated the average effect of adopting and dropping the Exam Playbook on their subsequent exams. Because most of the activity of Exam Playbook usage within a class occurred within the first two exams of the class (94%), we restricted this analysis to only the first two exams of each class. Stratified matching analysis was performed for each class separately (13 classes; the Introductory Economics Winter class did not have sufficient sample size for stratified matching) and we computed a meta-analytic estimate using a mixed-effects meta-analysis.

**Fig. 2:**
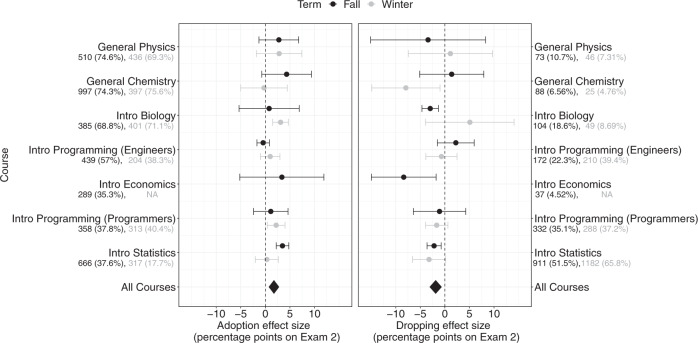
Meta-analysis of the Effect of Adopting and Dropping the Exam Playbook. Note. Forest plot showing effect sizes from stratified matching analyses. Numbers below each course name indicate the number of students in that analysis (and as a percentage of the total class). Left: Effect of “adopting” the Exam Playbook. Both groups did not use the Exam Playbook at Exam 1; students who used it on Exam 2 outperformed students who did not. Right: Effect of “dropping” the Exam Playbook. Both groups used the Exam Playbook for Exam 1; students who dropped the Exam Playbook at Exam 2 did worse than students who consistently used it. Error bars reflect 95% confidence intervals.

To estimate the average effect of adopting the Exam Playbook, we took the subset of students who did not use the Exam Playbook on their first exam. Of these, some students adopted the Exam Playbook on their second exam, while others did not. When matched on their first exam performance, college entrance scores, and demographics, students who adopted the Exam Playbook performed an average of 1.75 percentage points ([0.69, 2.81], *d* = 0.12, *p* = .001) better on the second exam, compared to those who never used it (Fig. 2 left panel).

To estimate the effect of dropping the Exam Playbook, we repeated this analysis on the subset of students who had used the Exam Playbook for their first exam. Of these students, some dropped the Exam Playbook on their second exam, while others continued using it. When matched on their first exam performance, college entrance scores, and demographics, students who dropped the Exam Playbook performed an average of 1.88 percentage points ([0.64, 3.11], *d* = 0.14, *p* = .003) *worse*, compared to those who kept using it (Fig. 2 right panel).

Following our earlier conservative test of generalizability beyond Introductory Statistics, repeating this stratified matching analyses with the 6 other courses excluding Introductory Statistics, we still observed these effects of adopting and dropping the Exam Playbook—albeit with smaller effect sizes. When matched on their first exam performance, college entrance scores, and demographics, students who adopted the Exam Playbook performed an average of 1.56 percentage points ([0.47 2.65], *d* = 0.10, *p* = .005 better on the second exam, compared to those who never used it. When matched on their first exam performance, college entrance scores, and demographics, students who dropped the Exam Playbook performed an average of −1.53 percentage points ([−3.29, 0.22], *d* = −0.12, *p* = .087) worse, compared to those who kept using it (although this smaller effect of dropping was not significant at the 0.05 level).

Overall, these intra-individual data add further evidence to our meta-analyses suggesting that, on average, using the Exam Playbook predicts exam performance. We describe in Supplementary Note 3 that these results also replicate using a difference-in-difference analytical method.

### Dosage and timing

Next, we examined whether there were dosage and timing effects of using the Exam Playbook. Uptake of the Exam Playbook peaked between the first two exams, and then dropped thereafter if there were more than 2 exams in the course (see Table 1). Mixed-effects meta-analyses indicated that using the Exam Playbook on more occasions (i.e., higher dosages) related to better average exam performance (*b* = 2.18 percentage points [1.18, 3.19], *d* = 0.18, *p* < 0.001) among students who used the Exam Playbook—consistent with findings from the original efficacy experiments^6^.

The Exam Playbook was made available to students up to 10 days prior to their exams. The average student who used the Exam Playbook engaged with it a week (*M* = 7.0 days, *sd* = 3.0 days) before their exams. We used time of usage (number of days before the exam) to predict exam performance at the exam-level. Students who used the Exam Playbook benefited more from using it earlier (*b* = 0.42 percentage points per day [0.29, 0.54], *d* = 0.03 per day, *p* < 0.001). This suggests that early preparation is associated with better Exam Playbook effectiveness, although it could also reflect other motivation-relevant traits like better time-management and general self-regulatory ability^34^. For example, students who used the Exam Playbook very close to the exam date might have procrastinated or crammed their exam preparation—reflecting lower self-regulation^35^.

### What kinds of students naturally used the exam playbook? Were there differential benefits to different groups of students?

To better understand which students naturally used the Exam Playbook as a learning resource, we ran a mixed-effects logistic regression using academic ability (college entrance exam score) and demographic variables (gender, race, first-generation status) as predictors of whether students used the Exam Playbook at least once in their classes. Academic ability did not significantly predict Exam Playbook usage (*χ*^2^(1) = 0.24, *p* = .621), which suggests that natural adoption of this Exam Playbook resource may not have been restricted to higher performers or simply more motivated students. However, there were demographic differences in natural uptake of the Exam Playbook. Gender significantly predicted Exam Playbook adoption (*χ*^2^(1) = 196.18, *p* < .001): the odds of females using the Exam Playbook were 2.22 times higher than males. Race also predicted Exam Playbook adoption (*χ*^2^(7) = 21.78, *p* = .003): in particular, Black and Hispanic students were less likely to use the Exam Playbook on their exams (Black students had 0.65 times the odds of using it compared to White students, *p* = .003, and 0.56 times the odds compared to Asian students, *p* < .001; Hispanic students had 0.79 times the odds of using it compared to White students, *p* = .026, and 0.68 times the odds of using it compared to Asian students, *p* < .001). First-generation status did not predict Exam Playbook adoption (*χ*^2^(1) = 0.79, *p* = .373).

Could certain groups of students have benefitted more (or less) from using the Exam Playbook? We fitted separate mixed-effects linear models to test the moderation effect of gender, race, and first-generation status on the effectiveness of using the Exam Playbook. Gender significantly moderated Exam Playbook effects: while females generally performed worse than males (*b* = −3.83 [−4.50, −3.17], *d* = 0.30, *p* < .001), as is commonly observed in STEM classes, female users benefitted 2.35 percentage points (*b* = 2.35 [1.45, 3.26], *d* = 0.19, *p* < .001) more from using the Exam Playbook than male users—a substantial 61.4% reduction in the gender gap. Race did not moderate Exam Playbook effects (*χ*^2^(7) = 6.11, *p* = .527). First-generation status significantly moderated Exam Playbook effects: while first-generation students generally performed worse than non-first-generation students (*b* = −7.04 [−7.95, −6.12], *d* = 0.57, *p* < .001), using the Exam Playbook reduced this gap by an average of 2.25 [0.96, 3.54], *d* = 0.18, *p* < .001, percentage points—a 32.0% reduction in the first-generation achievement gap.

## Discussion

Recent discussions on non-replications and lack of implementation fidelity when practitioners try to execute social-psychological interventions themselves^36–39^ suggest that more rigorous effectiveness tests are needed. Social-psychological interventions that target the social, affective, or motivational mechanisms of learning can be efficacious in rigorous laboratory or field trials, but still need to be further tested for their effectiveness when released for self- or facilitated-administration. Granted, not all social-psychological interventions are meant to be self-administered by students—but where they are and can be after distribution, it is worthwhile to systematically track and understand their use and benefits “in the wild.”

Our research provides an example of a large-scale, systematic effectiveness test of an efficacious intervention, addressing crucial empirical questions about its benefits, boundary conditions, users, and self-administered timing and dosage. We emphasize that such effectiveness research does not merely apply intervention design to practice in an atheoretical manner—instead, it importantly informs how scientists should think about intervention design and testing, along with the myriad factors that affect its translational effectiveness in actual classrooms (e.g., classroom climate, student demographics, timing, dosage). By identifying possible boundary conditions and other sources of intervention heterogeneity, this work is a step toward building better theories of the contextual factors and psychological mechanisms that matter for self-administered effectiveness—theories that future research could systematically test with additional measures of such contextual differences and psychological states^22,40^.

Building on earlier RCT causal evidence^6^, the purpose of this research was to scale, examine who takes up a freely available intervention resource, and to investigate its heterogeneity and generalizability. To minimize the limitations of drawing inferences from correlational data, we presented converging evidence on the potential benefits of Exam Playbook use from multiple analytical approaches—including estimating the meta-analytic effect size at both the class (*d* = 0.18) and exam levels (*d* = 0.22), a robustness test that controlled for prior academic performance (*d* = 0.14), stratified matching analysis (*d* = 0.12/0.13) for adding/dropping the Exam Playbook between exams, and difference-in-difference modeling (*d* = 0.16/0.12 for adding/dropping; Supplementary Note 3). The effects observed here (using both inter- and intra-individual modeling) are consistent with previous RCT evidence, showing that greater Exam Playbook usage relates to higher academic performance, even when controlling for obvious third variables.

Moreover, these observed benefits associated with Exam Playbook use were not simply due to students’ concurrent use of other learning resources available on ECoach (e.g., grade calculator, “to do list”^21^;). Other research on general ECoach use and engagement, conducted with a separate sample of students across 5 courses, found that using the Exam Playbook significantly and uniquely predicted course performance, even when controlling for the use of other ECoach resources^21^.

We tested for possible heterogeneity in the self-administered intervention effects, with a primary interest in understanding how the class climate might relate to students’ accrued benefits. Using the Exam Playbook seems to be more useful when more classmates use it and when teachers encourage its use—in other words, class norms supporting strategic resource use matter. Teachers could proactively encourage and nurture the psychology of strategic resource use in their courses—such as by incentivizing the use of the Exam Playbook, by encouraging groups of students to work together on the Exam Playbook, or by incorporating self-regulated resource use into their teaching.

There is also the possibility that the when individual students use the intervention, they learn to value and engage in self-regulated resource use to a greater degree. This intra-individual change can contribute to ecological change at the classroom level^31,40^, creating a learning environment with norms that value and support engagement in self-regulated resource use. Such bidirectional effects of intra-individual effects on classroom climate and classroom climate on students’ benefits are worth future investigation, because they shape how we understand where an intervention will effectively take root and how its effects might perpetuate^3^. Future research could also examine whether individual differences^41^, course structures, curricula, or demographic make-ups may be associated with greater (or less) intervention benefits.

Of secondary interest, we also tested for and discovered differences in Exam Playbook uptake by gender, race, and first-generation status. Compared to males, female students tend to be more conscientious, and may naturally be drawn toward organizing and planning their learning^42–45^, which the Exam Playbook facilitates. Hence, they also tend to benefit more from its use. These results suggest that Exam Playbook adoption could potentially help reduce the gender gap in STEM classes—an idea that intervention research should systematically investigate in an RCT. Although first-generation status did not predict Exam Playbook usage, first-generation student users did benefit more than non-first-generation students from using the resource—suggesting that we could encourage greater Exam Playbook adoption among first-generation students to promote their self-regulation and learning.

Black and Hispanic students were less likely than White and Asian students to use the Exam Playbook, even when it was freely available. It could be that these students experience greater identity threat in some of their STEM classes, which may undermine their motivation to engage in their classes and with resources provided for their learning^46^. Future research could pair threat-reducing interventions (such as values affirmation and belonging interventions) with the Exam Playbook to test if this might pave the way for greater use and benefits among these minority groups.

This research is among the first to follow an RCT-validated social-psychological intervention through effectiveness testing, after it is released for students’ self-administration. It demonstrates an example of successful scaling and generalizability of a class-tailored intervention; and highlights the importance of class climate, self-administered timing and dosage, and student background in explaining heterogeneity in uptake and benefits. We hope this will encourage even more effectiveness research at scale on how people adopt and benefit from social-psychological interventions, when given the free choice to use it or not.

## Methods

We adapted the Exam Playbook from the original Strategic Resource Use intervention, and delivered it using ECoach technology to multiple classes. ECoach enabled us to tailor its content (e.g., set of resources described, exam reminder) and delivery (timing of the intervention delivery before exams, total possible dosages offered) to each class. This study was approved as exempt from further oversight by the University of Michigan Institutional Review Board (IRB #HUM00119869). The research reported here was conducted as secondary data analysis, and under FERPA exception for educational research, given that the use of the ECoach platform (and the Exam Playbook feature) is now a standard part of the institution’s educational practices.

### ECoach technology for class-specific tailoring and delivering the intervention at scale

At our test university, ECoach technology is widely used (it currently has 24,165 users in 2021), and complements the university’s Learning Management System as a source of academic advising and various learning resources^20,21,47^. To deliver the Exam Playbook to the 14 STEM classes in our study, we leveraged this cutting-edge technology that was familiar and easily accessible to students.

The Exam Playbook was housed within ECoach as one of many available learning resources that students could choose to use (or not) autonomously. This approximates actual college learning, where students often have many resources (e.g., course packs, textbooks, peer study groups, library books, teacher office hours, online discussion forums) that they can choose to use or not for their learning^48^. This enabled us to test whether and how students naturally use the Exam Playbook when it is freely available as a learning resource, among many others, rather than when it is one specifically isolated resource imposed upon them in an RCT.

For each of the 14 STEM classes, psychologists, designers, and instructors collaborated to customize class-specific parts of the Exam Playbook, such as the checklist of available resources, and tailored exam reminders. As mentioned earlier, students received a personalized reminder via ECoach that the Exam Playbook was available before each of their course exams. This reminder was delivered on the online ECoach website, through email, or through text message, depending on the student’s notification preferences in ECoach.

ECoach automatically tracked and organized students’ (a) use of and responses to the Exam Playbook, (b) course exam performance data from the University’s Learning Management System, and (c) registrar data (e.g., prior performance, demographics). These data allowed us to test our research questions, described above.

### Exam playbook

Students were informed via ECoach personalized messaging that the Exam Playbook was available to them as an exam preparatory resource to use if they wished. As in previous RCTs^6^, access to the pre-exam exercise was officially made available 10 days prior to an exam. This was customized according to the timing and number of the exams in each course (see Supplementary Note 4 for more details about timing). For example, students in the Introductory Statistics course had the Exam Playbook available for use before each of their 3 exams, and they were sent a message via ECoach about this available resource 10 days before each exam. Importantly, students could decide for themselves whether or not to use the Exam Playbook, and this resource was provided alongside a list of other online learning resources on ECoach that were also freely available to students. To complement the earlier description of the Exam Playbook, we provide example screenshots of key components of the Exam Playbook in Supplementary Note 5. At the end of the Exam Playbook, students were offered a summary of their responses (including the resources they selected, their reasons why each resource would be useful, and their plans) to print out and keep if they chose.

### Courses

The 7 courses that were involved in our study across 2 consecutive semesters included: Introductory Statistics, Introductory Biology, General Chemistry, Introductory Economics, Introductory Programming (for Programmers), Introductory Programming (for Engineers), and General Physics. All except Introductory Economics are officially considered large introductory STEM courses.

### Statistical approach

Our analysis strategy involved computing effects within each of the 14 classes we observed (7 courses x 2 semesters), which themselves have between 1 to 4 exams. Then, treating each class as a separate “experiment”, we would compute a meta-analytic effect size using a random-effects meta-analysis model^30^. We took this general approach to all our analyses. Meta-analysis estimates were computed using the *meta* package (v4.18-1^49^) in *R* (see Supplementary Note 6 for replication using hierarchical linear modeling, and Supplementary Note 7 for R code for all of our models).

#### Treatment effect of exam playbook

For each class, we predicted students’ average exam performance using a binary predictor that indicated whether the student had used the Exam Playbook at least once in the class, operationalized as logging into the Exam Playbook and fully clicking through the complete intervention. We then aggregated the estimates from the 14 individual models, weighting them using their standard errors.

For the first robustness check, we added college entrance exam scores as a covariate. For the second robustness check, we repeated this analysis at the exam level. That is, we predicted exam score using a binary predictor whether the student used the Exam Playbook on that particular exam. We then aggregated the exam effects into a class effect, and then aggregated the effects across classes.

#### Class heterogeneity analysis

We predicted the Exam Playbook effect size of each class using the proportion of Exam Playbook usage in the class (i.e., proportion of students that used the Exam Playbook at least once, from 0 to 1) and a binary predictor indicating whether extra course credit was offered for using the Exam Playbook.

#### Stratified matching analysis

We performed stratified matching using the *MatchIt* package (v4.2.0^50^). Because of the steep drop-off in Exam Playbook usage after the first two exams, we focused on our analyses on Exam Playbook usage and exam performance on the first two exams (see Supplementary Note 8 for a discussion of this cut-off, including background, plausible explanations, and future directions). This analysis first computes a propensity score by using the covariates (previous exam score, college entrance score, gender, race, and first-generation status) to predict the treatment group (e.g., adopted the Exam Playbook versus not) via logistic regression. It then stratifies the propensity scores based on five quantiles. Based on these strata, the final regression model is weighted to give an estimate of the Average Treatment Effect (ATE) on the performance on the second exam. This analysis was run on each class separately. The aggregated estimated was computed via random-effects meta-analysis (using the *meta* package like above).

#### Dosage and timing

We fit linear models for each class before estimating an aggregate effect using random-effects meta-analysis. To estimate the dosage effect, we considered the subset of Exam Playbook users, and used the number of times they used the Exam Playbook to predict their average exam score in the class.

To estimate how timing of usage affects exam performance, we again considered the subset of Exam Playbook users, but now examined performance on each individual exam. We defined a variable, “time_left,” which counts the number of days between the Exam Playbook usage and the exam itself.

#### Moderation of Exam Playbook usage and effects

To test for self-selection, we predicted whether a student engaged with the Exam Playbook at least once in the class, using as predictors their college entrance scores, gender, race, and first-generation status. Similar to previous analyses, this analysis was performed separately for each class and aggregated using random-effects meta-analysis.

To estimate if the Exam Playbook effect size is moderated by gender, race, and first-generation status, we tested (separately in three models) the interaction of Exam Playbook usage with gender, race, and first-generation status. To compute first-generation status from the available registrar data, we classified students as “first-generation” if their parents had not received a college degree or above.

### Reporting summary

Further information on research design is available in the Nature Research Reporting Summary linked to this article.

## Supplementary information

Supplementary Information

Reporting Summary

## Data Availability

The data is protected under the Family Educational Rights and Privacy Act (FERPA) and any access to the underlying data is contingent on approval from the University of Michigan, per FERPA guidelines and regulations. Requests for student data should be sent to the Office of Enrollment Management at student.data.request@umich.edu.

## Code availability

Analysis code is available on Open Science Framework at https://osf.io/6qej7/^51^.

## References

[1] Walton GM, Yeager DS (2020). Seed and soil: psychological affordances in contexts help to explain where wise interventions succeed or fail. Curr. Directions Psychol. Sci..

[2] Hattie J (2009). Visible Learning: A synthesis of over 800 meta-analyses relating to achievement.

[3] Walton GM, Wilson TD (2018). Wise interventions: psychological remedies for social and personal problems. Psychol. Rev..

[4] Yeager DS, Walton GM (2011). Social-psychological interventions in education: they’re not magic. Rev. Educ. Res..

[5] Brady ST, Cohen GL, Jarvis SN, Walton GM (2020). A brief social-belonging intervention in college improves adult outcomes for Black Americans. Sci. Adv..

[6] Chen P, Chavez O, Ong DC, Gunderson B (2017). Strategic resource use for learning: a self-administered intervention that guides self-reflection on effective resource use enhances academic performance. Psychol. Sci..

[7] Cohen GL, Garcia J, Apfel N, Master A (2006). Reducing the racial achievement gap: a social-psychological intervention. Science.

[8] Paunesku D (2015). Mind-set interventions are a scalable treatment for academic underachievement. Psychol. Sci..

[9] Walton GM, Cohen GL (2011). A brief social-belonging intervention improves academic and health outcomes of minority students. Science.

[10] Yeager DS (2019). A national experiment reveals where a growth mindset improves achievement. Nature.

[11] Cohen J, McCabe L, Michelli NM, Pickeral T (2009). School climate: research, policy, practice, and teacher education. Teach. Coll. Rec..

[12] Miyake A (2010). Reducing the gender achievement gap in college science: a classroom study of values affirmation. Science.

[13] Kizilcec RF, Pérez-Sanagustín M, Maldonado JJ (2017). Self-regulated learning strategies predict learner behavior and goal attainment in Massive Open Online Courses. Comput. Educ..

[14] Kizilcec RF (2020). Scaling up behavioral science interventions in online education. Proc. Natl Acad. Sci. USA.

[15] Flay BR (1986). Efficacy and effectiveness trials (and other phases of research) in the development of health promotion programs. Prev. Med..

[16] Rosqvist, J., Thomas, J. C., & Truax, P. Effectiveness versus efficacy studies. In J. C. Thomas & M. Hersen (Eds.), *Understanding Research in Clinical and Counseling Psychology* (pp. 319–354). New York, NY: Routledge (2011).

[17] Bryk AS, Gomez LM, Grunow A, LeMahieu PG (2015). Learning to improve: How America’s schools can get better at getting better.

[18] Chen, P. The strategic resource use intervention. In G. M. Waltion, & A. J. Crum (Eds.), *Handbook of wise interventions: How social psychology can help people change* (pp. 166–190). Guilford Press. (2020).

[19] Gollwitzer PM (1999). Implementation intentions: strong effects of simple plans. Am. Psychol..

[20] Huberth M, Chen P, Tritz J, McKay TA (2015). Computer-tailored student support in introductory physics. PLoS ONE.

[21] Matz, R. et al. Analyzing the efficacy of ECoach in supporting gateway course success through tailored support. *LAK21: 11th International Learning Analytics and Knowledge Conference*, 216–225 (2021).

[22] Bryan CJ, Tipton E, Yeager DS (2021). Behavioural science is unlikely to change the world without a heterogeneity revolution. Nat. Hum. Behav..

[23] Hill CJ, Bloom HS, Black AR, Lipsey MW (2008). Empirical benchmarks for interpreting effect sizes in research. Child Dev. Perspect..

[24] Kraft MA, Blazar D, Hogan D (2018). The effect of teacher coaching on instruction and achievement: a meta-analysis of the causal evidence. Rev. Educ. Res..

[25] Silverman SL (2009). From randomized controlled trials to observational studies. Am. J. Med..

[26] Victora CG, Habicht J, Bryce J (2004). Evidence-based public health: moving beyond randomized trials. Am. J. Public Health (1971).

[27] Wasserstein RL, Lazar NA (2016). The ASA statement on p-values: context, process, and purpose. Am. Stat..

[28] Cumming G (2014). The new statistics: why and how. Psychol. Sci..

[29] Goh JX, Hall JA, Rosenthal R (2016). Mini meta‐analysis of your own studies: Some arguments on why and a primer on how. Soc. Personal. Psychol. Compass.

[30] Borenstein M, Hedges LV, Higgins JP, Rothstein HR (2010). A basic introduction to fixed‐effect and random‐effects models for meta‐analysis. Res. Synth. Methods.

[31] Powers JT (2016). Changing environments by changing individuals: the emergent effects of psychological intervention. Psychol. Sci..

[32] Bierman KL (2010). The effects of a multiyear universal social–emotional learning program: the role of student and school characteristics. J. Consulting Clin. Psychol..

[33] Austin PC (2011). An introduction to propensity score methods for reducing the effects of confounding in observational studies. Multivar. Behav. Res..

[34] Steel P (2007). The nature of procrastination: a meta-analytic and theoretical review of quintessential self-regulatory failure. Psychol. Bull..

[35] Carvalho PF, Sana F, Yan VX (2020). Self-regulated spacing in a massive open online course is related to better learning. npj Sci. Learn..

[36] Dweck, C., What having a “growth mindset” actually means. *Harvard Business Review*. (2016). Available online at: https://hbr.org/2016/01/what-having-a-growth-mindset-actually-means.

[37] Pelletier GN, Goegan LD, Chazan DJ, Daniels LM (2020). Agreeing is not the same as accepting: exploring pre-service teachers’ growth mindsets. Can. J. N. Scholars Educ./Rev. Canadienne des. Jeunes Chercheures et. Chercheurs en. Éducation.

[38] Walton GM, Crum AJ (2020). Handbook of wise interventions.

[39] Yeager DS, Dweck CS (2020). What can be learned from growth mindset controversies?. Am. Psychol..

[40] Binning KR, Browman AS (2020). Theoretical, ethical, and policy considerations for conducting social–psychological interventions to close educational achievement gaps. Soc. Issues Policy Rev..

[41] Chen P, Powers JT, Katragadda KR, Cohen GL, Dweck CS (2020). A strategic mindset: an orientation toward strategic behavior during goal pursuit. Proc. Natl Acad. Sci. USA.

[42] Keiser HN, Sackett PR, Kuncel NR, Brothen T (2016). Why women perform better in college than admission scores would predict: exploring the roles of conscientiousness and course-taking patterns. J. Appl. Psychol..

[43] Liu OL, Rijmen F, MacCann C, Roberts R (2009). The assessment of time management in middle-school students. Personal. Individ. Differences.

[44] Misra R, McKean M (2000). College students' academic stress and its relation to their anxiety, time management, and leisure satisfaction. Am. J. Health Stud..

[45] Virtanen P, Nevgi A (2010). Disciplinary and gender differences among higher education students in self‐regulated learning strategies. Educ. Psychol..

[46] Steele CM, Aronson J (1997). A threat in the air: how stereotypes shape intellectual identity and performance. Am. Psychol..

[47] Center for Academic Innovation. *Ecoach*. (2021) https://ai.umich.edu/software-applications/ecoach/.

[48] Chen P, Ong DC, Ng J, Coppola BP (2021). Explore, exploit, and prune in the classroom: strategic resource management behaviors predict performance. AERA Open.

[49] Balduzzi S, Rücker G, Schwarzer G (2019). How to perform a meta-analysis with R: a practical tutorial. Evid.-Based Ment. Health.

[50] Ho DE, Imai K, King G, Stuart EA (2011). MatchIt: nonparametric preprocessing for parametric causal inference. J. Stat. Softw..

[51] Chen et al. From Controlled Trials to Classrooms (Data Repository). Open Science Framework (2022) 10.17605/OSF.IO/6QEJ7.

