# Supplementary material for: Real-world effectiveness of a social-psychological intervention translated from controlled trials to classrooms
Source: NPJ Sci Learn. 2022 Aug 29;7:20. doi: 10.1038/s41539-022-00135-w (PMC9424297; doi:10.1038/s41539-022-00135-w)
Supplement: Supplementary file 1 — Supplementary Information [file 41539_2022_135_MOESM1_ESM.pdf]

Supplementary Information
for
Real-World Effectiveness of a Social Psychological Intervention Translated from Controlled Trials to Classrooms

Patricia Chen, Dennis W. H. Teo, Daniel X. Y. Foo, Holly A. Derry, Benjamin T. Hayward, Kyle W. Schulz, Caitlin Hayward, Timothy A. McKay, & Desmond C. Ong

Supplementary Table 1

Demographics of the students in our sample.

	Semester	
	**Fall Semester**	**Winter Semester**
**Gender**		
Male	3750 (54.4)	2582 (49.9)
Female	3137 (45.5)	2585 (49.9)
Not Indicated	2 (0.0)	9 (0.2)
**Academic Level**		
Freshman	2929 (42.5)	1118 (21.6)
Sophomore	2803 (40.7)	2752 (53.2)
Junior	828 (12.0)	921 (17.8)
Senior	307 (4.5)	366 (7.1)
Not Indicated	22 (0.3)	19 (0.4)
**Race**		
Caucasian	3908 (56.7)	3039 (58.7)
African American	268 (3.9)	215 (4.2)
Hispanic	490 (7.1)	371 (7.2)
Asian	1606 (23.3)	1015 (19.6)
Others	303 (4.4)	258 (5.0)
Not indicated	314 (4.6)	278 (5.4)
**Family Income**		
Less than US$50,000	833 (12.1)	653 (12.6)
US$50,000 - US$99,999	958 (13.9)	689 (13.3)
More than US$100,000	3308 (48.0)	2480 (47.9)
Not Indicated	1790 (26.0)	1354 (26.2)
**First-Generation Status**		
First-generation	885 (12.8)	709 (13.7)
Non-First-generation	5569 (80.8)	4146 (80.1)
Not Indicated	435 (6.3)	321 (6.2)
**Multi-enroll**	2182 (31.7)	1439 (27.8)

Note. Numbers in parentheses indicate percentage of total sample in that semester, to 1 decimal place. Multi-enroll indicates the number of students that were enrolled in more than one class (of the seven we studied) in that semester.

Supplementary Table 2

Descriptives of the total number and percentages of (i) students who adopted the Exam Playbook, compared to (ii) students who never used the Exam Playbook; and (iii) students who dropped the Exam Playbook, compared to (iv) students who consistently used the Exam Playbook.

		Number of students who adopted the Exam Playbook (E1 Not Used, E2 Used)	Number of students who never used the Exam Playbook (E1 Not Used, E2 Not Used)	Number of students who dropped the Exam Playbook (E1 Used, E2 Not Used)	Number of students who consistently used the Exam Playbook (E1 Used, E2 Used)
Intro Statistics	Fall	403 (22.9%)	330 (18.7%)	124 (7.0%)	904 (51.3%)
	Winter	141 (7.9%)	235 (13.1%)	263 (14.6%)	1157 (64.4%)
Intro Biology	Fall	26 (4.6%)	416 (74.3%)	83 (14.8%)	35 (6.3%)
	Winter	204 (36.2%)	294 (52.1%)	17 (3.0%)	49 (8.7%)
General Chemistry	Fall	50 (3.7%)	1179 (88.4%)	91 (6.8%)	14 (1.0%)
	Winter	51 (9.8%)	440 (84.6%)	18 (3.5%)	11 (2.1%)
General Physics	Fall	38 (5.6%)	560 (81.9%)	76 (11.1%)	10 (1.5%)
	Winter	163 (25.9%)	413 (65.7%)	24 (3.8%)	29 (4.6%)
Intro Programming (Engineers)	Fall	116 (15.1%)	436 (56.6%)	86 (11.2%)	132 (17.1%)
	Winter	94 (17.6%)	187 (35.1%)	68 (12.8%)	184 (34.5%)
Intro Programming (Programmers)	Fall	144 (15.2%)	333 (35.2%)	88 (9.3%)	381 (40.3%)
	Winter	150 (19.4%)	239 (30.8%)	77 (9.9%)	309 (39.9%)
Intro Economics	Fall	12 (2.6%)	392 (85.2%)	46 (10.0%)	10 (2.2%)
	Winter	3 (0.8%)	336 (94.5%)	12 (3.4%)	3 (0.8%)

Note. The sample size in Intro Economics Winter semester was too small for some analyses, hence also the large error bar in the main text Figure 1 forest plot.

Supplementary Note 1

Additional Details on Definition and Operationalization of Exam Playbook "Use"

As described in the main text, we operationalized a "use" of the Exam Playbook to mean accessing and completing the intervention, including: completing the resource checklist, explaining why each resource would be useful, and planning resource use. Students had to click through to the end of the intervention to be counted as having used it.

In the table below, we detail how many instances there were of students who started using the Exam Playbook, and how many of those students finished it. For some classes, such as both Intro Programming classes, and Intro Statistics, over 83% of students who started the resource finished it. For other classes, the completion rates were lower, ranging from 30%-65%. In this paper, we counted only instances where students completed the Exam Playbook as a "use."

Supplementary Table 3

Descriptives of the number of instances where students started the Exam Playbook and the number (and percentage) of those who completed using it, categorized by course and semester

		Number of instances across all exams where student started the Exam Playbook	Completed Exam Playbook (considered as "used")
**Intro Statistics**	Fall	4135	3760 (90.9%)
	Winter	4388	4088 (93.2%)
**Intro Biology**	Fall	497	240 (48.3%)
	Winter	753	485 (64.4%)
**General Chemistry**	Fall	596	186 (31.2%)
	Winter	233	119 (51.0%)
**General Physics**	Fall	370	144 (38.9%)
	Winter	484	316 (65.3%)
**Intro Programming (Engineers)**	Fall	544	466 (85.7%)
	Winter	593	530 (89.4%)
**Intro Programming (Programmers)**	Fall	1178	994 (84.4%)
	Winter	1013	845 (83.4%)
**Intro Economics**	Fall	255	115 (45.1%)
	Winter	83	27 (32.5%)

Supplementary Note 2

Description of Selected Resources

While the resource checklist were tailored based on the respective classes, we observed that students gravitated to similar types of resources across courses. The table below presents the top 5 selected resources (by Exam Playbook users) within each class, as well as the proportion of instances in which these resources were selected. From these rankings, we observed the following common types of resources that were consistently popular across classes: 1) practice problems (e.g., problem roulette, practice exams; popular in 12 out of 14 classes); 2) lecture content and notes (e.g., lecture notes, lecture slides; popular in 10 classes); and 3) exam review sessions (popular in 8 classes). While noting that some courses have unique resources (e.g., Videos on Flip It Physics, formula card), these trends suggest that there was, in general, consistency in the types of learning resources that Exam Playbook users tended to choose across classes.

Top 5 Most Popular Selected Resources by Class

Course	Fall	Percentage	Winter	Percentage
**Intro Statistics**	Practice exam questions (on Canvas)	94.28	Practice exam questions (on Canvas)	58.46
	Lecture notes	71.97	Lecture notes	53.28
	Problem Roulette past exam questions	71.44	Past required homework problems	48.95
	Formula card	60.35	Past recommended homework problems	48.92
	Past required homework problems	59.02	Problem Roulette past exam questions	47.63
**Intro Biology**	Doing practice exams (from Canvas)	94.58	Doing practice exams (from Canvas)	91.34
	Doing practice quizzes (from Launchpad)	80.42	Doing practice quizzes (from Launchpad)	74.85
	Doing practice homework sets (from Canvas)	79.17	Doing practice homework sets (from Canvas)	72.16
	Practicing concept mapping and retrieval	58.33	Writing answers to coursepack learning objectives	49.48

	Reorganize textbook and lecture notes together	57.92	Reorganize textbook and lecture notes together	46.19
**General Chemistry**	Problem Roulette practice problems	86.02	Problem Roulette proactice problems	79.83
	Extra practice questions in OWL	77.96	Lecture notes	75.63
	Exam review session (in lecture)	68.82	Exam review session (in lecture)	60.5
	ECoach exam tips	66.13	Extra practice questions in OWL	60.5
	Lecture notes	62.37	Extra practice questions in textbook	47.06
**General Physics**	Problem Roulette practice problems	88.19	Practice exams (on Canvas)	95.89
	Lecture notes (on Canvas)	74.31	Problem Roulette practice problems	70.57
	ECoach exam tips	70.83	Lecture notes (on Canvas)	53.8
	Readings from the textbook	65.97	Readings from the textbook	50
	Videos on Flip It Physics	58.33	Questions, exercises, & problems from the textbook	49.68
**Intro Programming (Engineers)**	Review my labs	83.69	Review my projects	86.98
	Lecture slides (on Google Drive)	83.05	Review my labs	85.09
	Review my projects	81.12	Lecture slides (on Google Drive)	81.51
	Review Weekly Review questions	67.81	Review Weekly Review questions	65.85
	Exam review session (in lecture)	53.65	Exam review session (in lecture)	55.09
	Past exams (on Canvas)	85.61	Past exams (on Canvas)	87.46
	zyBooks readings	76.16	zyBooks readings	78.82

**Intro Programming (Programmers)**	183Study past exam questions	73.04	Exam review	74.91
	Exam review	72.43	183Study past exam questions	74.2
	CodeLab exercises	64.08	CodeLab exercises	68.64
**Intro Economics**	Practice problems	96.52	Practice problems	100
	Exam review session (in lecture)	81.74	Lecture slides	85.19
	Lecture slides	81.74	Exam review session (in lecture)	81.48
	ECoach exam tips	61.74	Rewriting my lecture notes	66.67
	Rewriting my lecture notes	56.52	ECoach exam tips	40.74

Note. The percentage refers to the proportion that the resource was selected over the total number of opportunities given (or number of times the Exam Playbook was used).

Supplementary Note 3

Difference-in-difference Analysis

An alternative method of assessing the effect of adopting or dropping the Exam Playbook is using a difference-in-differences (DiD) regression model (Angrist & Pischke, 2008). Here, we report our results using this model and show that it replicates our results obtained by stratified matching that were reported in the main text.

Similar to our analysis using stratified matching, we restricted our analyses to only the first two exams of each class. To estimate the effect of adopting the Exam Playbook, we took the subset of students who did not use the Exam Playbook on their first exam. For each class, we ran a separate DiD model, controlling for college entrance scores, gender, race, and first-generation status, and aggregated the regression estimates using a random-effects meta-analysis. After students adopted the Exam Playbook, they performed better on the subsequent exam by an average of 2.04 percentage points ([0.81, 3.26], $d = 0.16$, $p = .001$). We repeated this analysis to estimate the effect of dropping the Exam Playbook, by taking the subset of students who used the Exam Playbook on their first exam. Controlling for college entrance scores, gender, race, and first-generation status, we estimated that after dropping the Exam Playbook, students performed worse by 1.80 percentage points ([-3.15, -0.44], $d = -0.12$, $p = .009$). These estimates were consistent in terms of general direction and magnitude with the estimates from our analyses using stratified matching (1.75 percentage points, $d = 0.12$ for adopting and -1.88 percentage points, $d = -0.14$ for dropping).

If we exclude Introduction to Statistics to test the generalization of the Exam Playbook, we find that the difference-in-difference analysis still yields a significant positive effect of adoption. Controlling for college entrance scores, gender, race, and first-generation status, students who adopted the Exam Playbook performed better on the subsequent exam by an average of 1.75 percentage points ([0.27, 3.22], $d = 0.14$, $p = 0.021$). However, excluding Introductory Statistics, we found that the difference-in-difference effect for dropping the playbook is not statistically significant at the 0.05 level ($b = 0.38$ percentage points ([-1.59, 2.35], $d = 0.03$, $p = 0.705$).

Specifically, these were the models we ran on each class. To estimate the effect of adopting the Exam Playbook, we ran:

```
lm(exam_score ~ adopted_playbook*time + college_entrance_score + gender + race + first_gen, data = subset(exam_lvl, did_not_use_playbook_on_exam1))
```

This analysis only includes the students who did not use the Exam Playbook in the first exam. “adopted_playbook” is a dummy-coded variable that indicates the students who started using the Exam Playbook on their second exam.

To estimate the effect of dropping the Exam Playbook, we ran:

```
lm(exam_score ~ dropped_playbook*time + college_entrance_score + gender + race + first_
gen, data = subset(exam_lvl, used_playbook_on_exam1))
```

This analysis only includes the students who used the Exam Playbook in the first exam. "dropped_playbook" is a dummy-coded variable that indicates the students who dropped the Exam Playbook on their second exam.

Supplementary Note 4

Additional Information About Administration Timing

Due to logistical errors in communication between the intervention administration team and instructors, 137 (1.1% out of 12,065) students were accidentally given access to the Exam Playbook earlier than 10 days prior to their exams. Because the planned official release date was 10 days prior to the exam, and this was also the earliest timing that the vast majority of students could access the Exam Playbook via ECoach, in the main paper analysis we report analyses using a truncated "time_left" variable that ensured values fell between 0-10 (i.e., any value above 10 was replaced with 10). Nevertheless, we also repeated this analysis without truncation (i.e., using 15 days before the exam that was the maximum time that any student had accessed the Exam Playbook). Consistent with the main findings, students who used the Exam Playbook benefitted more from using it earlier (b = 0.42 percentage points per day [0.29, 0.54], $p < .001$; compared to b = 0.42 percentage points per day without truncation).

Supplementary Note 5

Screenshots of Key Components of the Exam Playbook

Selection of Resources from a (Class-Specific) Resource Checklist

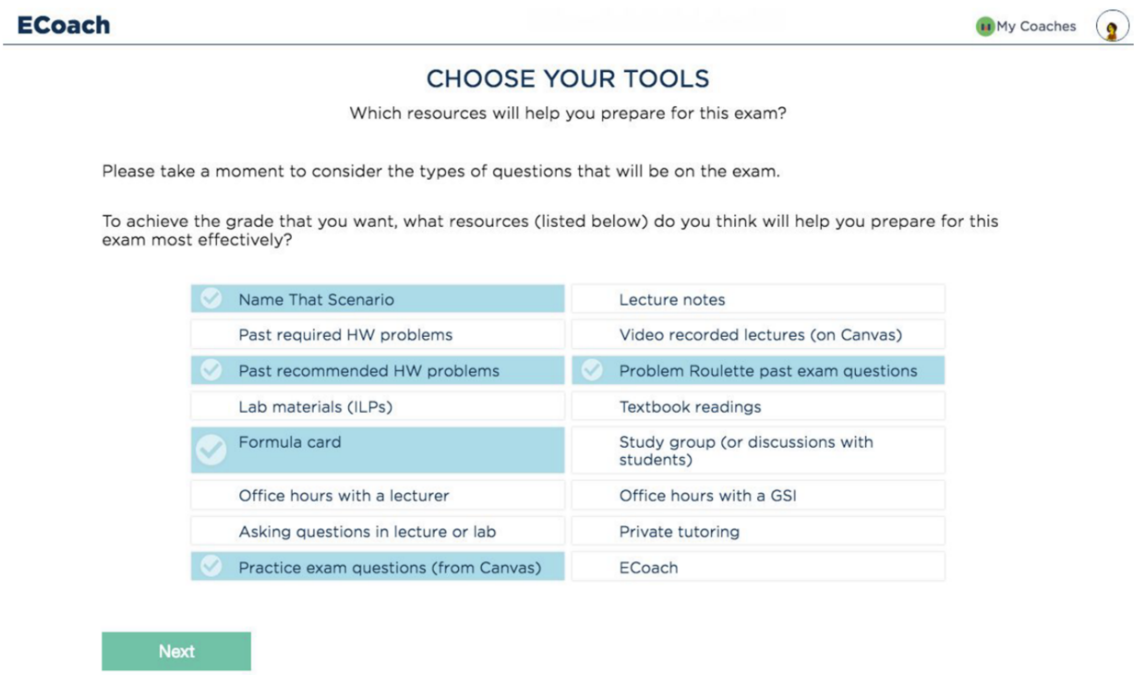

Explanation of Why Each Resource Would be Useful for Learning

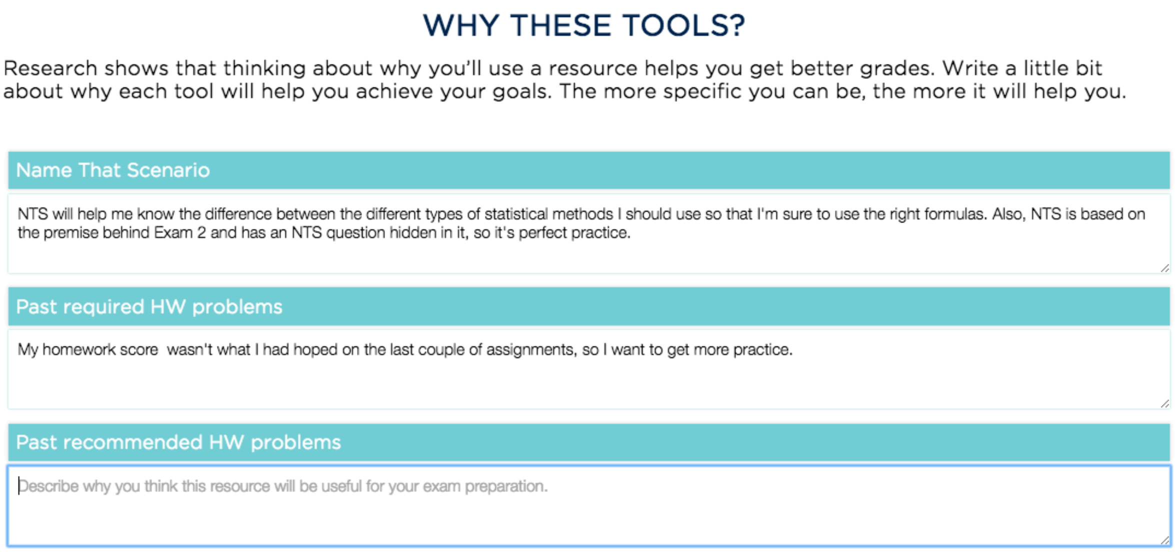

Planning When, Where, and How to Use The Chosen Resources

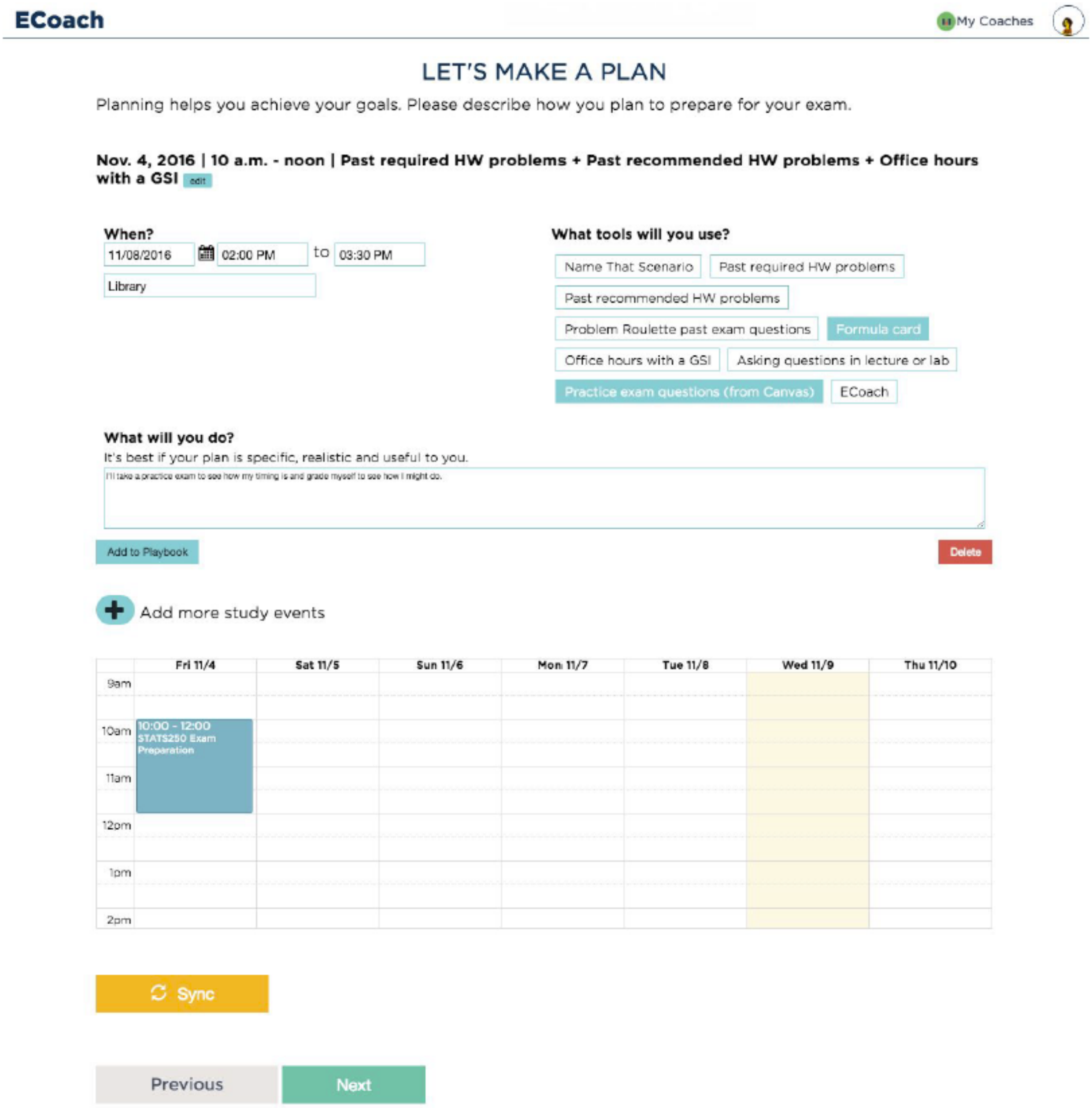

Supplementary Note 6

Mixed-effects Hierarchical Linear Modelling

In our analyses in the main text, we used a mixed-effects meta-analysis model to aggregate the effect size estimates across the different classes, treating each class as a separate “experiment”. We preferred this approach as we wanted to further examine heterogeneity across classes. An alternative analysis approach is mixed-effects hierarchical linear modelling, where we treat students as nested within course and semester. Here, we report our results using this alternative approach, using the lme4 package (v1.1-26; Bates et al., 2015), of estimation and show that we can draw similar conclusions.

Effect of Using the Exam Playbook. To estimate the effect of using the Exam Playbook, we used a dummy-coded variable indicating that a student used the Exam Playbook at least once throughout the semester (playbook_user) to predict their average exam score in the class. We added random effects by course and semester (Note: We tried fitting a model with course nested within semester, but the model reported a singular fit, suggesting that the random-effect structure is over-fitted.). Specifically, we ran the following model:

```
lmer(avg_exam_score ~ playbook_user + (1|course) + (1|semester), data= user_lvl)
```

Consistent with the meta-analysis model, we found that students who used the Exam Playbook outperformed students who did not (b = 2.07 percentage points [1.51, 2.64], d = 0.11, $p < .001$; compared to 2.17 percentage points, d = 0.18, estimated by meta-analysis). We did a further robustness check to repeat this analysis at the exam level:

```
lmer(exam_score ~ used_playbook + (1|exam:course) + (1|student:course) +
    (1|semester), data= exam_lvl)
```

We found that students who used the Exam Playbook on a given exam performed better than students who did not (b = 2.94 percentage points [2.60, 3.28], d = 0.12, $p < .001$; compared to 2.91 percentage points, d = 0.22, estimated by meta-analysis).

Dosage and Timing. To estimate the dosage effect, we considered the subset of Exam Playbook users, and used the number of times they used the Exam Playbook to predict their average exam score in the class. We added random effects by course and semester.

```
lmer(avg_exam_score ~ sum_playbook_usage + (1|course) + (1|semester),
    data= playbook_users)
```

We found that among students who used the Exam Playbook, using the Exam Playbook on more occasions related to better average exam performance (b = 3.33 percentage points [2.90, 3.76], d = 0.26, $p < .001$; compared to b = 2.18, d = 0.18, estimated via meta-analyses).

To estimate how timing of usage affects exam performance, we again considered the subset of Exam Playbook users, but now examined performance on each individual exam. We defined a variable, "time_left", which counts the number of days between the Exam Playbook usage and the exam itself. We used this to predict students' exam score. Because this was at the exam level (which is nested within course and semester), we used the following random effect structure:

```
lmer(exam_score ~ time_left + (1|exam:course:semester) + (1|course) + (1|semester), data= p
laybook_users_exam_level)
```

We found that students who used the Exam Playbook benefited more from using it earlier (b = 0.53 percentage points per day [*95% CI*: 0.46, 0.61], d = 0.39, p < .001; compared to b = 0.42, d = 0.03, estimated via meta-analyses).

Supplementary Note 7

R Code Corresponding to Main Text Analyses

Treatment effect of Exam Playbook. For each class, we predicted students' average exam performance using a binary predictor that indicated whether the student used the Exam Playbook at least once in the class. We then aggregated the estimates from the 14 individual models, weighting them using their standard errors.

```
playbook_effect <- lm(avg_exam ~ used_playbook, data=class_data)
... # repeat for all classes, merge into all_playbook_effects
meta::metagen(all_playbook_effects$estimates, all_playbook_effects$se,
comb.random = TRUE)
```

Class Heterogeneity Analysis. We predicted the Exam Playbook effect size of each class using the proportion of Exam Playbook usage in the class (i.e., proportion of students that used the Exam Playbook at least once, from 0 to 1), and a binary predictor indicating whether extra course credit was offered for using the Exam Playbook.

```
lm(playbook_effect_size ~ proportion_playbook_use + course_credit, data =
all_classes)
```

Stratified Matching Analysis. We performed stratified matching using the *MatchIt* package (v4.2.0; Ho et al., 2011). This analysis first computes a propensity score by using the covariates (previous exam score, college entrance score, gender, race, and first-generation status) to predict the treatment group (e.g., adopted the Exam Playbook versus not) via logistic regression. It then stratifies the propensity scores based on quantile. Based on these strata, the final regression model is weighted to give an estimate of the Average Treatment Effect (ATE). Below, usage_pattern is a categorical variable that could be "adopted" versus "not adopted", or "dropped" versus "not dropped".

```
matched_data = match.data(
        matchit(usage_pattern ~ E1_exam_score + college_entrance_score +
gender + race + first_gen, data=class_data,
                method = "subclass",
                subclass = 5, # 5 strata
                estimand = "ATE"))

lm(E2_exam_score ~ usage_pattern + E1_exam_score + college_entrance_score
+ gender + race + first_gen, weights = weights, data = matched_data)
```

Dosage and Timing. We fit linear models for each class before estimating an aggregate effect using random-effects meta-analysis. To estimate the dosage effect, we considered the

subset of Exam Playbook users, and used the number of times they used the Exam Playbook to predict their average exam score in the class.

```
lm(avg_exam_score ~ sum_playbook_usage, data= playbook_users)
```

To estimate how timing of usage affects exam performance, we again considered the subset of Exam Playbook users, but now examined performance on each individual exam. We defined a variable, "time_left," which counts the number of days between the Exam Playbook usage and the exam itself.

```
lm(exam_score ~ time_left, data= playbook_users_exam_level)
```

Moderation of Exam Playbook usage and effects. To test for self-selection, we predicted whether a student used the Exam Playbook at least once in the course, using as predictors their college entrance scores, gender, race, and first-generation status. Similar to previous analyses, this analysis was performed separately for each class and aggregated using random-effects meta-analysis.

```
glm(used_playbook ~ college_entrance_score + gender + race + first_gen,
data=user.lvl, family = "binomial")
```

To estimate if the Exam Playbook effect size is moderated by gender, race, and first-generation status, we tested (separately in three models) the interaction of Exam Playbook usage with race, gender, and first-generation status:

```
lm(avg_exam_score ~ used_playbook*gender, data=user.lvl)

lm(avg_exam_score ~ used_playbook*race, data=user.lvl)

lm(avg_exam_score ~ used_playbook*first_gen, data=user.lvl)
```

Supplementary Note 8

Focus on the First Two Exams in Stratified Matching Analysis

As described in the main text, we performed stratified matching analysis on Exam Playbook usage and exam performance in the first two exams, due to the steep drop-off in Exam Playbook usage after the first two exams across the majority of courses (with the exception of the introductory statistics course; see Table 1). This analytic decision mirrored analysis of the original RCT intervention dosage, where students were only given the intervention up to two times (Chen et al., 2017). As background, the original intervention was developed for a introductory college statistics course with only two exams. Offering two doses was considered enough to convey the message and yet not too much repetition to bore students. This translational study differed by providing as many doses as students voluntarily wanted to self-administer (for example, some classes allowed students to take the Exam Playbook before each of their 3 exams, such that students who did not want to stop at two doses but preferred to use the Exam Playbook more were given the opportunity to do so).

As the usage rates in Table 1 show, however, there was generally a steep drop in students' usage of the Exam Playbook after the first two exams across most courses (with the exception of the introductory statistics course which incentivized the use of the Exam Playbook on the third exam). This steep drop-off in use could mean different things: one, that students get tired of repeatedly seeing the same intervention content and no longer use it after two tries; or two, that students have understood and internalized the psychology of the intervention, and hence no longer need it further.

To elaborate on the first possibility, because each dosage of the intervention is the same in content, offering too many doses of the exact same intervention may bore students, resulting in dropping usage rates beyond two doses. Future iterations of the Exam Playbook that plan to offer students more than two possible doses should examine this (such as through student focus groups or user experience interviews), and improve upon the intervention design.

The latter point on internalization is an important open empirical question that we are keen to further investigate in future research. Can students internalize and generalize the psychology of strategic resource use to their other classes and long-term? Longitudinal research with students' course and performance data could follow up on these results to study how much students truly internalize and transfer the psychology of the Exam Playbook.

Supplementary References

Angrist, J. D., & Pischke, J. S., *Mostly harmless econometrics*. Princeton University Press (2008).

Bates, D., Mächler, M., Bolker, B., & Walker, S., Fitting Linear Mixed-Effects Models Using lme4. *Journal of Statistical Software*, *67*(1), 1–48, (2015).

Chen, P., Chavez, O., Ong, D. C., & Gunderson, B., Strategic resource use for learning: A self-administered intervention that guides self-reflection on effective resource use enhances academic performance. *Psychological Science, 28*(6), 774-785, (2017).

Ho, D. E., Imai, K., King, G., & Stuart, E. A., MatchIt: Nonparametric preprocessing for parametric causal inference. *Journal of Statistical Software, 42*(8), (2011). https://doi.org/10.18637/jss.v042.i08
