# Supplementary material for: Real-world effectiveness of a social-psychological intervention translated from controlled trials to classrooms
Source: NPJ Sci Learn. 2022 Aug 29;7:20. doi: 10.1038/s41539-022-00135-w (PMC9424297; doi:10.1038/s41539-022-00135-w)
Supplement: Supplementary file 2 — Reporting Summary [file 41539_2022_135_MOESM2_ESM.pdf]

nature portfolio

Corresponding author(s): Patricia Chen
Last updated by author(s): May 25, 2022

Reporting Summary

Nature Portfolio wishes to improve the reproducibility of the work that we publish. This form provides structure for consistency and transparency in reporting. For further information on Nature Portfolio policies, see our Editorial Policies and the Editorial Policy Checklist.

Statistics

For all statistical analyses, confirm that the following items are present in the figure legend, table legend, main text, or Methods section.

n/a	Confirmed	
☐	☒	The exact sample size (*n*) for each experimental group/condition, given as a discrete number and unit of measurement
☐	☒	A statement on whether measurements were taken from distinct samples or whether the same sample was measured repeatedly
☐	☒	The statistical test(s) used AND whether they are one- or two-sided<br>*Only common tests should be described solely by name; describe more complex techniques in the Methods section.*
☐	☒	A description of all covariates tested
☒	☐	A description of any assumptions or corrections, such as tests of normality and adjustment for multiple comparisons
☐	☒	A full description of the statistical parameters including central tendency (e.g. means) or other basic estimates (e.g. regression coefficient) AND variation (e.g. standard deviation) or associated estimates of uncertainty (e.g. confidence intervals)
☐	☒	For null hypothesis testing, the test statistic (e.g. *F*, *t*, *r*) with confidence intervals, effect sizes, degrees of freedom and *P* value noted<br>*Give P values as exact values whenever suitable.*
☒	☐	For Bayesian analysis, information on the choice of priors and Markov chain Monte Carlo settings
☐	☒	For hierarchical and complex designs, identification of the appropriate level for tests and full reporting of outcomes
☐	☒	Estimates of effect sizes (e.g. Cohen's *d*, Pearson's *r*), indicating how they were calculated

Our web collection on statistics for biologists contains articles on many of the points above.

Software and code

Policy information about availability of computer code

Data collection	Data was collected on the ECoach platform, which is developed and maintained by the Center for Academic Innovation at the University of Michigan. More description of the ECoach platform can be found in previously published research (e.g., https://doi.org/10.1371/journal.pone.0137001, https://dl.acm.org/doi/abs/10.1145/3448139.3448160).
Data analysis	Analysis was done using R, which is open-source. The packages used (e.g., lmer, metagen) were all cited with version numbers, and sample syntax was also provided in the Supplemental Information. Full analysis code has been uploaded to a public OSF repository at https://osf.io/6qej7/ (as mentioned in the Code Availability)

For manuscripts utilizing custom algorithms or software that are central to the research but not yet described in published literature, software must be made available to editors and reviewers. We strongly encourage code deposition in a community repository (e.g. GitHub). See the Nature Portfolio guidelines for submitting code & software for further information.

Data

Policy information about availability of data

All manuscripts must include a data availability statement. This statement should provide the following information, where applicable:

- Accession codes, unique identifiers, or web links for publicly available datasets
- A description of any restrictions on data availability
- For clinical datasets or third party data, please ensure that the statement adheres to our policy

The data is protected under the Family Educational Rights and Privacy Act (FERPA) and any access to the underlying data is contingent on approval from the University of Michigan, per FERPA guidelines and regulations. Requests for student data should be sent to the Office of Enrollment Management at student.data.request@umich.edu.

Field-specific reporting

Please select the one below that is the best fit for your research. If you are not sure, read the appropriate sections before making your selection.

- [ ] Life sciences
- [x] Behavioural & social sciences
- [ ] Ecological, evolutionary & environmental sciences

For a reference copy of the document with all sections, see nature.com/documents/nr-reporting-summary-flat.pdf

Behavioural & social sciences study design

All studies must disclose on these points even when the disclosure is negative.

Study description	Quantitative study
Research sample	12,065 undergraduates from 14 STEM classes at a public Midwestern university in the USA. Detailed student demographics are provided in Table S1 in the SOM.
Sampling strategy	This was an analysis of a self-selected sample of students who used (vs. did not use) the educational technology resource. Hence, there was no a priori sampling criteria.
Data collection	We used ECoach technology to scale up, distribute, and track students' voluntary use of an online intervention called the Exam Playbook.
Timing	Fall 2017 - Winter 2018
Data exclusions	N/A. We used all available data from the students who interacted with ECoach in the 14 classes.
Non-participation	N/A. This was an analysis of a sample of students who used vs. did not use the online intervention on their own in 14 classes.
Randomization	N/A. No randomization.

Reporting for specific materials, systems and methods

We require information from authors about some types of materials, experimental systems and methods used in many studies. Here, indicate whether each material, system or method listed is relevant to your study. If you are not sure if a list item applies to your research, read the appropriate section before selecting a response.

Materials & experimental systems

n/a	Involved in the study	
☒	☐	Antibodies
☒	☐	Eukaryotic cell lines
☒	☐	Palaeontology and archaeology
☒	☐	Animals and other organisms
☐	☒	Human research participants
☒	☐	Clinical data
☒	☐	Dual use research of concern

Methods

n/a	Involved in the study	
☒	☐	ChIP-seq
☒	☐	Flow cytometry
☒	☐	MRI-based neuroimaging

Human research participants

Policy information about studies involving human research participants

Population characteristics	See above.
Recruitment	Students in 14 classes were given the free choice to use or not use a freely available online intervention, the Exam Playbook. Their use (or non-use) was tracked via an ECoach online system that they voluntarily accessed. We analyzed such use or non-use data that the ECoach system tracked.
Ethics oversight	University of Michigan

Note that full information on the approval of the study protocol must also be provided in the manuscript.
